# Proteomics Analysis of Tears and Saliva From Sjogren’s Syndrome Patients

**DOI:** 10.3389/fphar.2021.787193

**Published:** 2021-12-07

**Authors:** Nabangshu Das, Nikhil G. Menon, Luiz G. N. de Almeida, Paige S. Woods, Miriam L. Heynen, Gregory D. Jay, Barbara Caffery, Lyndon Jones, Roman Krawetz, Tannin A. Schmidt, Antoine Dufour

**Affiliations:** ^1^ Departments of Physiology and Pharmacology and Kinesiology, University of Calgary, Calgary, AB, Canada; ^2^ McCaig Institute, University of Calgary, Calgary, AB, Canada; ^3^ Department of Biomedical Engineering, School of Dental Medicine, UConn Health, Farmington, CT, United States; ^4^ Department of Emergency Medicine, Warren Alpert Medical School and School of Engineering, Brown University, Providence, RI, United States; ^5^ Centre for Ocular Research and Education (CORE), School of Optometry and Vision Science, University of Waterloo, Waterloo, ON, Canada; ^6^ Toronto Eye Care, Toronto, ON, Canada; ^7^ Department of Cell Biology and Anatomy, University of Calgary, Calgary, AB, Canada; ^8^ Hotchkiss Brain Institute, University of Calgary, Calgary, AB, Canada

**Keywords:** lubricin, PRG4, protease, protease inhibitor, proteomics, saliva, tears, Sjogren's Syndrome

## Abstract

Sjogren’s syndrome (SS) is characterized by dysfunctional mucous membranes and dysregulated moisture-secreting glands resulting in various symptoms, including dry mouth and dry eyes. Here, we wanted to profile and compare the tear and saliva proteomes of SS patients to healthy controls. Tear and saliva samples were collected and subjected to an isotopic dimethylation labeling shotgun proteomics workflow to identify alterations in protein levels. In tear samples, we identified 83 upregulated and 112 downregulated proteins. Pathway enrichment analysis of the changing proteins by Metascape identified leukocyte transendothelial migration, neutrophil degranulation, and post-translation protein phosphorylation in tears of SS patients. In healthy controls’ tears, an enrichment for proteins related to glycolysis, amino acid metabolism and apoptotic signaling pathway were identified. In saliva, we identified 108 upregulated and 45 downregulated proteins. Altered pathways in SS patients’ saliva included cornification, sensory perception to taste and neutrophil degranulation. In healthy controls’ saliva, an enrichment for proteins related to JAK-STAT signaling after interleukin-12 stimulation, phagocytosis and glycolysis in senescence were identified. Dysregulated protease activity is implicated in the initiation of inflammation and immune cell recruitment in SS. We identified 20 proteases and protease inhibitors in tears and 18 in saliva which are differentially expressed between SS patients and healthy controls. Next, we quantified endogenous proteoglycan 4 (PRG4), a mucin-like glycoprotein, in tear wash and saliva samples via a bead-based immune assay. We identified decreased levels of PRG4 in SS patients’ tear wash compared to normal samples. Conversely, in saliva, we found elevated levels of PRG4 concentration and visualized PRG4 expression in human parotid gland *via* immunohistological staining. These findings will improve our mechanistic understanding of the disease and changes in SS patients’ protein expression will help identify new potential drug targets. PRG4 is among the promising targets, which we identified here, in saliva, for the first time.

## Introduction

Sjogren’s syndrome (SS) is a chronic autoimmune disease that predominantly affects middle-aged women, with a high female to male ratio (9:1), and is characterized by both local and systemic inflammations, with exocrine glands (i.e., salivary, parotid and lacrimal glands) being the primary site of disease manifestation (Virdee et al., 2017; Vivino, 2017; Mavragani and Moutsopoulos, 2020). Lymphocyte infiltration and inflammation in salivary and lacrimal glands result in dryness of the mucosal surfaces, including the mouth (xerostomia) and eye (xerophthalmia), which are the key symptoms that lead to clinical suspicion of the disease occurring in more than 95% of the patients (Ramos-Casals et al., 2012; Brito-Zerón et al., 2016). Dry mouth and eye can progress into further complications, including accelerated caries, loss of dentition, corneal ulcer, vision loss, in addition to internal organ inflammation and morbidity (Vivino, 2017). SS is further defined as primary Sjogren’s syndrome (pSS) when it presents alone and as secondary Sjogren’s syndrome (sSS) when accompanied by other diseases, such as systemic lupus erythematosus (SLE), rheumatoid arthritis (RA) or systemic sclerosis (Mavragani and Moutsopoulos, 2020). Although the exact etiology of SS is not clear, previous research indicates that it is a multifactorial disease with involvement of both genetic and environmental factors activating innate and adaptive immune pathways (Ramos-Casals et al., 2012; Both et al., 2017; Mavragani, 2017). Around 75% of infiltrating lymphocytes in the salivary glands of SS constitute T cells, which are mostly composed of CD4^+^ T cells (Fox et al., 1983; Verstappen et al., 2017). These cells can produce both Th1 and Th2 cytokines but are characterized by a shift in the favor of Th1 in the salivary gland in patients with SS (Mitsias et al., 2002). Moreover, an overexpression of the cytokines IL4, IL17, and IFNγ have been associated with the pathogenesis of SS (Verstappen et al., 2017; López-Villalobos et al., 2021).

Annual healthcare costs for SS patients were found to be over twice those for a community control group and comparable to those for patients with RA (Callaghan et al., 2007). Unfortunately, diagnosis of SS is often delayed or incorrect due to our limited understanding of disease pathogenesis; therefore, a better characterization of disease mechanisms and pathways altered in SS will be beneficial. Salivary gland biopsy, which is often considered to be the gold standard test for SS, is invasive and can be associated with serious post-operative complications including excessive bleeding, swelling and numbness in the lower lip (Richards et al., 1992). Hallmarks of SS-associated markers include autoantibodies, such as anti-SSA and anti-SSB, IFNγ and other cytokines; however, these markers are also associated with other autoimmune diseases such as RA, SLE, or polymyositis and are therefore not specific to SS (Burbelo et al., 2009; Dufour et al., 2018; Seror et al., 2021). Furthermore, drugs previously implemented in other autoimmune diseases including RA and SLE failed to reach primary outcomes in randomized double-blind controlled trials for the treatment of SS (Mavragani and Moutsopoulos, 2020; Seror et al., 2021). Failure in therapeutic approaches targeting Cathepsin S, an inducible costimulator of T cell ligand and lymphotoxin beta receptor indicated lack of appropriate drug targets/specific biomarker in SS (Mavragani and Moutsopoulos, 2020). Therefore, a specific, sensitive, and non-invasive biomarker for SS diagnosis is urgently needed to reduce morbidity and improve patients’ quality of life.

Mass spectrometry (MS) is widely utilized as an effective proteomics tool for biomarker discovery owing to its ability to provide sensitive and selective detection, multi-analyte analysis, and information about post-translational modifications of proteins or peptides (Addona et al., 2009; Aebersold and Mann, 2016; Geyer et al., 2017). Advantages of MS and proteomics analyses is the capacity for a comprehensive characterization of proteomes using a small volume of tears (Zhou et al., 2009; Li et al., 2015) and saliva samples (Ryu et al., 2006; Al-Tarawneh et al., 2011). Importantly, collection of both tear and saliva samples is non-invasive and relatively simple to undertake; therefore, a routine collection could be done that could help in early diagnosis, monitoring disease progression, or treatment response. However, the precise mechanism and altered proteins have not been extensively characterized in SS patients.

Proteoglycan 4 (PRG4), also known as lubricin, is a mucin-like glycoprotein with an important role in maintaining homeostasis through its ability to provide boundary lubrication and regulate inflammatory signaling (Das et al., 2019). PRG4 expression in the eye is found to be critical for ocular surface composition (Schmidt et al., 2013; Samsom et al., 2015) and maintaining tear film integrity (Rabiah et al., 2020), as evidenced by the *Prg4* ^*−/−*^ mice that displayed significantly higher red corneal fluorescein staining (representing damage of the ocular surface) compared to wild type counterparts (Schmidt et al., 2013). A recent clinical trial showed that recombinant human PRG4 (rhPRG4) was effective at reducing signs and symptoms of dry eye disease, where ∼38% of the people enrolled were SS patients (Lambiase et al., 2017). *In vitro,* the expression of PRG4 by human corneal epithelial cells was found to be altered by the proinflammatory cytokines TNFα and IL1β (Menon et al., 2021). Furthermore, PRG4 was shown to be reduced on the corneal epithelium and in the lacrimal gland in an animal model of experimental dry eye disease (Menon et al., 2021). Despite some studies that have characterized certain protein changes in tears (Zhou et al., 2009; Li et al., 2015), it is still unclear what are the normal levels of PRG4 found in SS tears. Similar analyses have been performed in saliva, but PRG4 has never been detected (Ryu et al., 2006; Al-Tarawneh et al., 2011). Previously, we demonstrated that Cathepsin S, whose activity is upregulated in SS tear (Hamm-Alvarez et al., 2014; Hargreaves et al., 2019), is capable of proteolytic processing of endogenous PRG4 in tear samples as well as purified rhPRG4, that latter of which resulted in reduced ocular surface boundary lubricating properties (Regmi et al., 2017). Therefore, we wanted to conduct an unbiased investigation to identify what other proteases and proteases inhibitors could be implicated in the pathogenesis of SS. We also wanted to measure the levels of PRG4 in SS tears and saliva as a SS is characterized by dryness of mucosal surfaces like eyes and mouth. Here, using quantitative proteomics and bioinformatics analyses, we characterize tears and saliva biopsies in SS and healthy controls. Additionally, for the first time, we present evidence that PRG4 is downregulated in SS tear washes but upregulated in SS saliva as measured by quantitative proteomics and our custom PRG4 AlphALISA. We present new unbiased and quantitative proteomics data to improve our understanding of the mechanisms and altered proteins in SS patients.

## Materials and Methods

### Patient Information and Sample Collection

We were exempt from ethics for the analysis done as all samples were commercially purchased or from de-identified patients and from biobank indicated below. Tears and washes were collected from Sjogren’s syndrome (SS) patients and healthy controls (Table 1). All collection procedures were performed at the Centre for Ocular Research and Education, University of Waterloo, Ontario, Canada. SS patients were identified using the following criteria: Ocular Surface disease Index (OSDI) ≥ 23, non-invasive tear breakup time (NIBUT) < 10 s, and diagnosis of primary SS using the American European Consensus Criterion (Vitali et al., 2002). Healthy controls were identified using the following criteria: OSDI score <13, NIBUT ≥10 s, and has not been diagnosed with dry eye and does not use artificial tears, gels, or rewetting drops to relieve ocular symptoms. To collect tear, up to 5 µl of tears were collected from the inferior temporal tear meniscus, without corneal anesthesia, using a glass microcapillary tube. A maximum of 5 min of tear collection was allowed per eye. Tears were collected from both eyes and stored at −80°C. In total, tears were collected from 22 SS patients (20 female, 2 male, 60.0 ± 16.5 years old, mean ± SD) and 20 healthy patients (13 female, 7 male, 31.2 ± 11.4 years old, mean ± SD) (Table 1 and Supplementary Material).

**TABLE 1:** Patient’s information.

		Healthy controls		Sjogren’s syndrome	
**Tears**	Number	20		17	
	Age	31.2 ± 11.4 years old		56.2 ± 16.7 years old	
	Sex	13 females	7 males	15 females	2 males
**Tear Washes**	Number	29		14	
	Age	34.1 ± 14.2 years old		59.5 ± 12.0 years old	
	Sex	17 females	12 males	13 females	1 male
**Saliva**	Number	10		30	
	Age	46.8 ± 14.5 years old		45.2 ± 14.6 years old	
	Sex	5 females	5 males	22 females	8 males

The same criteria were used to select SS patients and healthy controls for collecting tear washes. Tears were collected from both eyes and stored at −80°C. To collect tear washes, 40 µl of sterile 0.9% saline was instilled onto the superior bulbar region of the eyes. The patients then rotated their eyes, and the washes were collected from the inferior fornix regions and stored separately at −80°C for each eye. In total, tear washes were collected from 14 SS patients (13 female, 1 male, 59.5 ± 12.0 years old, mean ± SD) and 29 healthy patients (17 female, 12 male, 34.1 ± 14.2 years old, mean ± SD) (Table 1 and Supplementary Material).

Unstimulated whole saliva samples from SS patients were obtained from the Sjogren’s International Collaborative Clinical Alliance (SICCA) biorepository (Table 1). SS patients were identified using the new American College of Rheumatology Classification Criteria for Sjogren’s syndrome (Shiboski et al., 2012). Patients had to be above 18 years old and recruited in the United States, as well as have at least two of the following criteria: positive serology for anti-SSA and/or anti-SSB antibodies (or positive rheumatoid factor and antinuclear antibodies (ANA) titer ≥1:320), ocular staining ≥3, and presence of focal lymphocytic sialadentis with a focus score ≥1 focus/4 mm^2^. Saliva samples from healthy controls were purchased from BioIVT (Westbury, NY). In total, 30 SS samples (22 female, 8 male, 45.2 ± 14.6, mean ± SD) and 10 healthy (5 female, 5 male, 46.8 ± 14.5, mean ± SD) were obtained (Table 1 and Supplementary Material). For the proteomics experiment, the closest age matched groups were selected: SS 37.8 ± 5.8 years old and healthy controls 42.2 ± 17.9 years old. We analyzed 5 female samples for SS and 2 females/3 males for healthy controls.

Parotid gland samples were obtained from the University of Connecticut Health Center’s Research Tissue Biorepository Core Facility, which were flash frozen in liquid nitrogen, embedded in optimal cutting temperature (OCT), and cryopreserved. In total, 5 samples were analyzed from four patients: two men, age 69- and 70- years old and two women, age 73- and 93-year-old. All patients all free of any cancer diagnosis at time of sample collection.

### Shotgun Proteomics Analysis

Tears and saliva were used for shotgun proteomics analysis. Protein samples were lysed with 1% SDS, 0.1 M EDTA in 200 nM HEPES (pH 8), protease cOmplete™ inhibitor tablets (Sigma-Aldrich, ON, Canada). Proteins were denatured with the addition of a final concentration of 10 mM dithiothreitol (DTT). Samples were alkylated by incubation with a final concentration of 15 mM iodoacetamide (IAA) in the dark for 25 min at room temperature. Samples were next digested with Trypsin (Promega, Madison, WI, United States ). With HCl the pH adjusted to 6.5. Next, to label peptide α- and ε-amines, samples were incubated for 18 h at 37°C with isotopically heavy (40 mM ^13^CD_2_O+20 mM NaBH_3_CN (sodium cyanoborohydride)) or light labels (40 mM light formaldehyde (CH_2_O) + 20 mM NaBH_3_CN), all final concentrations. Samples were subjected to C18 chromatography using Pierce™ C18 columns (Thermo Scientific™, ON, Canada) before being subjected to liquid chromatography and tandem mass spectrometry (LC-MS/MS).

### High Performance Liquid Chromatography (HPLC) and Mass Spectrometry (MS)

All liquid chromatography and mass spectrometry experiments were carried out by the Southern Alberta Mass Spectrometry (SAMS) core facility at the University of Calgary, Canada. Analysis was performed on an Orbitrap Fusion Lumos Tribrid mass spectrometer (Thermo Scientific, ON, Canada) operated with Xcalibur (version 4.0.21.10) and coupled to a Thermo Scientific Easy-nLC (nanoflow Liquid Chromatography) 1,200 system. Tryptic peptides (2 μg) were loaded onto a C18 trap (75 um x 2 cm; Acclaim PepMap 100, P/N 164,946; Thermo Scientific, ON, Canada) at a flow rate of 2 μl/min of solvent A (0.1% formic acid and 3% acetonitrile in LC-MS grade water). Peptides were eluted using a 120 min gradient from 5 to 40% (5–28% in 105 min followed by an increase to 40% B in 15 min) of solvent B (0.1% formic acid in 80% LC-MS grade acetonitrile) at a flow rate of 0.3 μl/min and separated on a C18 analytical column (75 um x 50 cm; PepMap RSLC C18; P/N ES803; Thermo Scientific). Peptides were then electrosprayed using 2.3 kV voltage into the ion transfer tube (300°C) of the Orbitrap Lumos operating in positive mode. The Orbitrap first performed a full MS scan at a resolution of 120,000 FWHM to detect the precursor ion having an *m*/*z* between 375 and 1,575 and a +2 to +7 charge. The Orbitrap AGC (Auto Gain Control) and the maximum injection time were set at 4 × 10^5^ and 50 ms, respectively. The Orbitrap was operated using the top speed mode with a 3 s cycle time for precursor selection. The most intense precursor ions presenting a peptidic isotopic profile and having an intensity threshold of at least 5,000 were isolated using the quadrupole and fragmented with HCD (30% collision energy) in the ion routing multipole. The fragment ions (MS^2^) were analyzed in the ion trap at a rapid scan rate. The AGC and the maximum injection time were set at 1 × 10^4^ and 35 ms, respectively, for the ion trap. Dynamic exclusion was enabled for 45 s to avoid the acquisition of the same precursor ion having a similar m/z (±10 ppm).

### Proteomic Data Analysis

Spectral data were matched to peptide sequences in the human UniProt protein database using the Andromeda algorithm (Cox et al., 2011) as implemented in the MaxQuant (Cox and Mann, 2008) software package v.1.6.10.23, at a peptide-spectrum match FDR of < 0.01. Search parameters included a mass tolerance of 20 p.p.m. for the parent ion, 0.5 Da for the fragment ion, carbamidomethylation of cysteine residues (+57.021464 Da), variable N-terminal modification by acetylation (+42.010565 Da), and variable methionine oxidation (+15.994915 Da). N-terminal and lysine heavy (+34.063116 Da) and light (+28.031300 Da) dimethylation were defined as labels for relative quantification. The cleavage site specificity was set to Trypsin/*p* for the proteomics data, with up to two missed cleavages allowed. Significant outlier cutoff values were determined after log (2) transformation by boxplot-and-whiskers analysis using the BoxPlotR tool (Spitzer et al., 2014). The data were deposited into ProteomeXchange via the PRIDE database and are freely available (PXD028922).

### Bioinformatics Analysis

To identify protein–protein interactions, the STRING (Search Tool for the Retrieval of Interacting Genes) database was used to illustrate interconnectivity among proteins (Szklarczyk et al., 2019). Protein-protein interactions relationship were encoded into networks in the STRING v11 database (https://string-db.org). Data were analyzed using the *homo sapiens* as the organism at a false discovery rate of 5% and the reactome pathways were analyzed. Metascape analysis was also used to identify enriched pathways (Zhou et al., 2019). Protein-protein interactions relationship were encoded into networks using the metascape website (https://metascape.org/), and the enriched pathways were plotted as heatmaps. The Circos graphs were generated with Metascape. On the outside, each arc represents the identity of each gene list (Sjogren’s syndrome in magenta and Healthy control in grey). On the inside, each cyan arc represents a gene list, where each gene has a spot on the arc. Black lines link the different genes where they connect to the same ontology term. A large number of black lines indicates higher amount of functional overlap among the input gene lists.

### Histology

Five different OCT blocks from four different patients were sectioned onto slides and probed for immunoreactivity to anti-PRG4 mAb 4D6 (Abubacker et al., 2016) (Regmi et al., 2017) using an anti-mAb-HRP conjugate developed with DAB and counter stained with light hematoxylin. Negative control samples received no primary mAb (University of Connecticut Health Center Research Histology Core).

### PRG4 Quantification in Tear Wash and Saliva

PRG4 levels in saliva and tear washes were quantified using a bead-based immunoassay using the AlphaLISA^®^ (Perkin-Elmer, ON, Canada) platform technology. Full-length rhPRG4 (Lubris BioPharma) was biotinylated (brhPRG4) using a commercially available kit (EZ-Link Sulfo-NHS-LC-Biotinylation Kit, Thermo Scientific 21,435), as per the manufacturer’s instructions. Anti-PRG4 mAb 4D6 (Regmi et al., 2017) was bound to AlphaScreen unconjugated acceptor beads (Perkin-Elmer 6,762,003), following manufacturer guidelines. Then, 5 μl of brhPRG4 per well (at 8 ng/ml) was mixed with 5 μl per well of 4D6-conjugated AlphaLISA acceptor beads at a final concentration of 80 μg/ml and 5 μl per well of streptavidin coated AlphaScreen donor beads (Perkin-Elmer 6760002S) at a final concentration of 80 μg/ml. Next, 15 μl of the resulting solution was transferred to each well of an opaque 96-well half-plate under low light. Finally, 5 μl of rhPRG4 (at concentrations of 0.8, 8, 80, 240, 800, 8,000, 80,000 μg/ml) or 5 μl of tear/saliva sample at multiple serial dilutions were added to the wells and allowed to incubate for 2 h protected from light. The total reaction volume summed to 20 μl, including 5 μl of sample, with final concentrations of 1 ng/ml, 20 μg/ml, and 20 μg/ml for the brhPRG4, 4D6-conjugated acceptor beads, and streptavidin coated donor beads, respectively. Plates were then read on a SpectraMax i3 microplate reader (Molecular Devices) using an excitation wavelength of 680 nm and an emission wavelength of 625 nm. All reagents were prepared and/or diluted in PBS with 0.05% Tween 20, and all incubations were done at room temperature, with gentle nutation, and a plate sealer to prevent evaporation. All samples were run in duplicate. Calculated PRG4 levels were also normalized to total protein concentrations measured using a BCA assay (Thermo Scientific 23,227).

### PIGNON Analysis

For PIGNON analysis, the normalized protein ratios were used as calculated by Maxquant and available in the proteingroups. txt file. PIGNON was set to use the “physical link” network (v11) from the STRING database due to the small size of our dataset. The mapping file was obtained from the input_file.zip available at PIGNON’s Github (https://github.com/LavalleeAdamLab/PIGNON). The inferred Gene Ontology (GO) terms were obtained from www.git.dhimmel.com/gene-ontology. The STRING accepted combined score was set to 400. The tear and saliva datasets were analyzed with weighted and unweighted networks. The number of samplings was set to 100,000. The sampling type was set to weighted. The Monte Carlo sampling was set to 3 and 500 for the lower and upper bound, respectively. The results included mapped pathways that were trimmed according to an FDR of 1%. Next, only the GO terms unique to the weighted network were considered true positives, which were obtained by subtracting the GO terms that were also present in the unweighted network. The lists of enriched GO terms, along with the *p*-values, were submitted to Revigo (Supek et al., 2011) to summarize the biological functions and provide visualization of enriched terms. The scatterplot and table generated by Revigo was modified using R (v4.0.3) (R Development Core Team, 2011) to substitute the color scale with a magenta-based color palette.

### Statistical Analysis

The differences in PRG4 concentration, volume, and mass in tear washes were analyzed, using the Prism software, using Welch’s unpaired 2-way *t*-tests if the data were heteroscedastic or unpaired 2-way t-tests otherwise. The difference in PRG4 concentration in saliva was analyzed using a Welch’s unpaired 2-way *t*-test. All data reported as mean ± standard deviation (SD).

## Results

### Quantitative Proteomics of Human Sjogren’s Syndrome Tear

**FIGURE 1:**
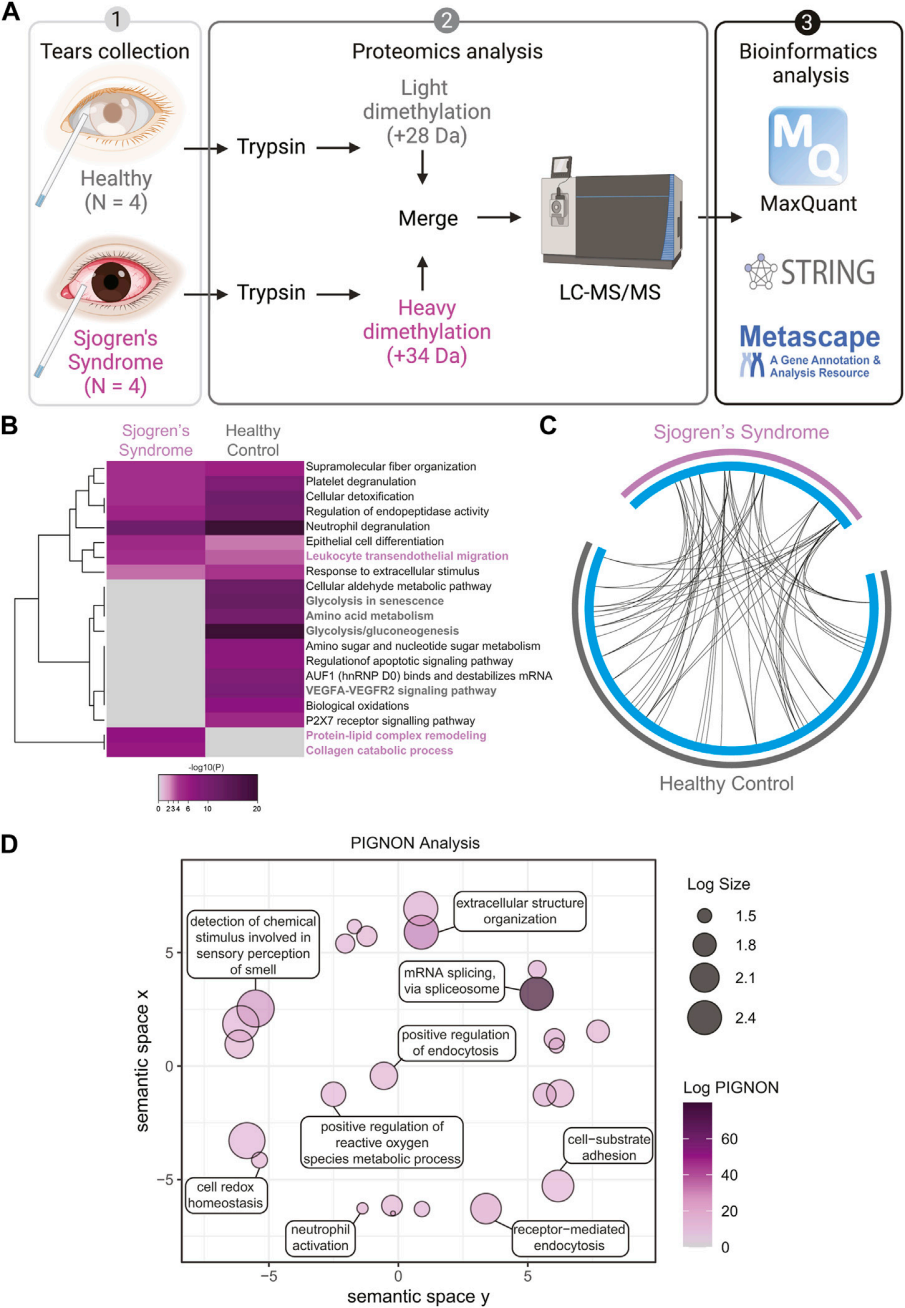
Quantitative Proteomics of Human Sjogren’s Syndrome Tears Washes. **(A)** Proteomics workflow and analysis (N = 4, Sjogren’s syndrome (SS) and N = 4 healthy controls). STRINGdb and Metascape software were used to identify pathway enrichment and protein-protein interactions. A list of all detected proteins is provided in Supplementary Table S1. **(B)** Metascape analysis of different pathways between SS and healthy control tear washes. Magenta marked are upregulated in SS and grey marked are upregulated pathways in healthy controls. Accumulative hypergeometric *p*-values and enrichment factors were calculated and used for filtering. Remaining significant terms were then hierarchically clustered into a tree based on Kappa-statistical similarities among their genes memberships. Then, 0.3 kappa score was applied as the threshold to cast the tree into term clusters. **(C)** Circos plot shows how significant proteins from the input lists overlap and were generated with Metascape. On the outside, each arc represents the identity of each gene list (Sjogren’s syndrome in magenta and Healthy control in grey). On the inside, each cyan arc represents a gene list, where each gene has a spot on the arc. Black lines link the different genes where they fall into the same ontology term. **(D)** Visualization of PIGNON results using Revigo. The x- and *y*-axis have no intrinsic meaning. In the plot, semantically similar GO terms remain close together. LogSize equals to the Log_10_(Number or annotations for GO Term ID in *Homo sapiens* in the EBI GOA database). Log PIGNON equals to the -Log_10_(FDR-adjusted *p* value as generated by PIGNON). The PIGNON analysis was performed using the normalized protein ratios as calculated by Maxquant. PIGNON was set to use the “physical link” network (v11) from the STRING database, due to the small size of our dataset. The Monte Carlo sampling was set to 3 and 500 for the lower and upper bound, respectively. The results included mapped pathways that were trimmed according to a false discovery rate (FDR) of 1%. The lists of enriched gene ontology (GO) terms, along with the *p*-values, were submitted to Revigo to summarize the biological functions and provide visualization of enriched terms.

To assess the global proteome changes in Sjogren’s syndrome (SS) patients, we collected tears of 4 SS patients and 4 healthy controls using glass microcapillary tube, and we performed a quantitative shotgun proteomics analysis (Figure 1A). Tear lysates were digested with trypsin and isotopically labeled. Healthy control tears were tagged with light formaldehyde (+28 Da) and SS patients’ tears with heavy formaldehyde (+34 Da; Figure 1A). Data were analyzed using MaxQuant (Cox and Mann, 2008) (Cox et al., 2011) at a 1% false discovery rate (FDR), and data integration for pathway and gene ontology (GO) enrichment was performed with Metascape (Zhou et al., 2019), STRING-db (Szklarczyk et al., 2019) and PIGNON (Nadeau et al., 2021). For the data interpretation, we describe changes in abundance of proteins as log2 fold change (SS tears over healthy controls), which means log2 values > 0 represent proteins that were upregulated in SS tears, <0 represents downregulation. In the proteomics analysis (Supplementary Figure S1 and Supplementary Table S1), we identified 83 unique proteins that were upregulated in SS tears and 112 unique downregulated proteins. By using the online meta-analysis tool Metascape (https://metascape.org/), we identified several enriched pathways between SS and healthy control tears (Figure 1B, Supplementary Figure S2A,B). Some enriched pathways in SS tears include leukocyte transendothelial migration (CTSB, ITGB2, MMP9), protein-lipid complex remodeling (PSAP, PLTP, APOE, MPO) and collagen catabolic process (CST3, VIM, MMP8, TGM2, ITGB2, MMP9, CTSB) (Figure 1B, Supplementary Table S1). Conversely, some enriched pathways in the healthy control tears include glycolysis/gluconeogenesis and glycolysis in senescence (ENO1, ENO2, ALDOA, PGD, PGK1, PGAM1), amino acid metabolism (MDH2, IDH1, ALDH1A1, GLO1, PKM) and VEGFA-VEGFR2 signaling pathway (HSP90AA1, PRDX2, PRDX6, RAN) (Figure 1B, Supplementary Table S1). Although we identified key differences in pathway enrichment and GO terms, there was some overlap between the SS and healthy control tears (Figure 1C).

**FIGURE 2:**
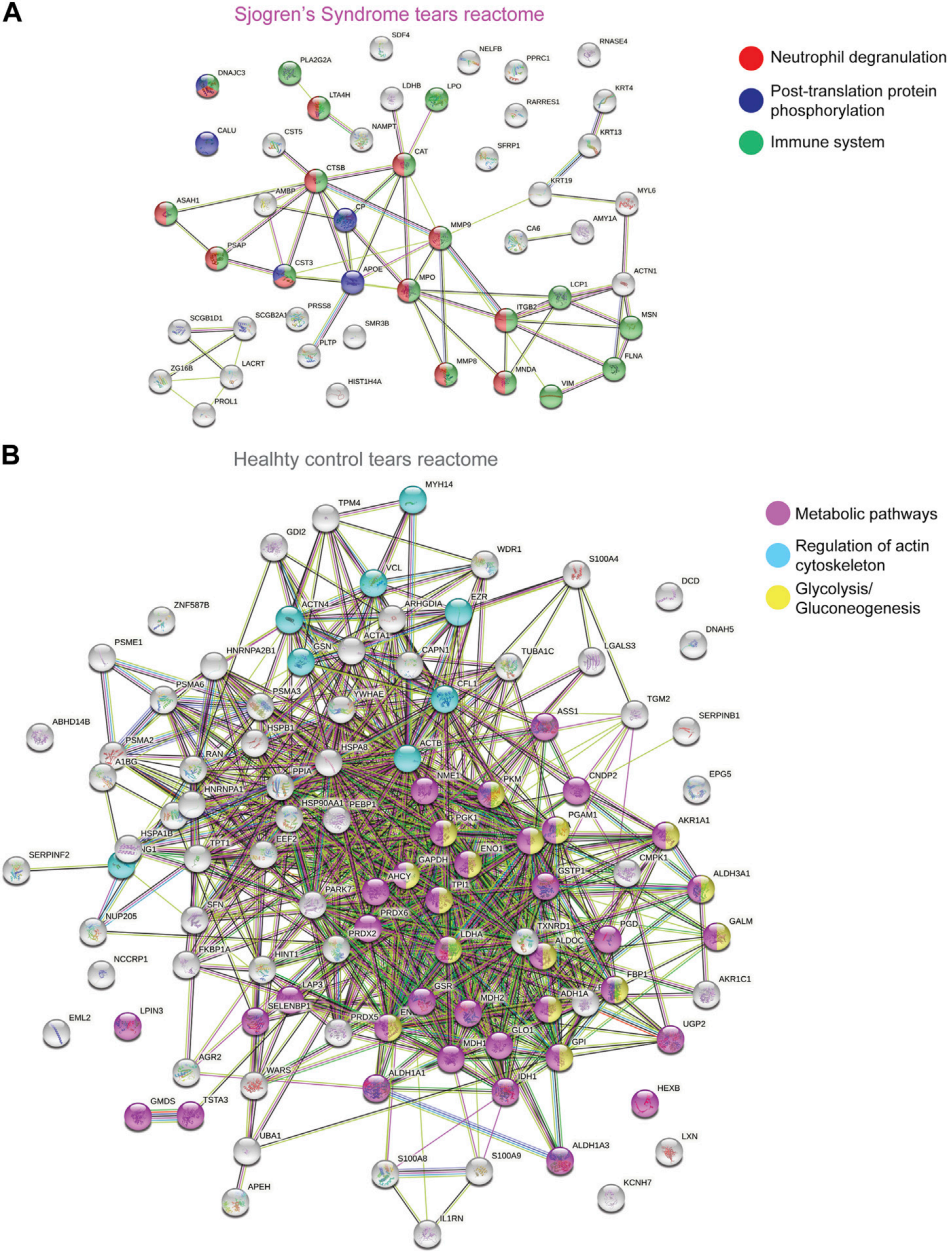
STRING Analysis of Quantitative Proteomics Data of Human Sjogren’s Syndrome Tears Washes. Analysis of protein−protein interaction network by STRING v11. Elevated proteins from **(A)** SS or **(B)** healthy control tears identified from the quantitative proteomics analysis were mapped by searching the STRING v11 software with a confidence level of 1% false discovery rate. Colored lines between the proteins indicate different types of interaction evidence: known interactions (teal), experimentally determined (pink), predicted interactions gene neighborhood (green), gene fusions (red), gene co-occurrence (blue), text-mining (yellow), coexpression (black), protein homology (purple).

To better understand and visualize our data, we used two additional bioinformatics tools, PIGNON (Nadeau et al., 2021) and STRING-db (Szklarczyk et al., 2019), to add a more comprehensive characterization of our tear washes dataset. PIGNON was used to identify enriched biological processes via GO terms, while STRING-db was used to identity reactome pathways. Using PIGNON, we identified mRNA splicing (via spliceosome), cell-substrate adhesion and detection of chemical organization stimulus involved in sensory perception of smell (Figure 1D, Supplementary Figure S2C). Using STRING-db v11 (https://string-db.org), we observed that the majority of the upregulated proteins in SS tears were proteins clustered with neutrophil degranulation as red nodes (CTSB, ITGB2, MMP8, MMP9), post-translation protein phosphorylation as blue nodes (APOE, CALU, CP, CST3) and immune system as green nodes (CTSB, ITGB2, LCP1, MMP8, MMP9) (Figure 2A). In healthy control tears, enriched reactome pathways include metabolic pathways as purple nodes (ENO1, ENO2, ALDOA, PGD, PGK1, PGAM1), regulation of actin cytoskeleton as cyan nodes (ACTB, ACTN4, CFL1, EZR, VCL) and glycolysis/gluconeogenesis as yellow nodes (ALDH3A1, ALDOC, ENO1, FBP1, PKM) (Figure 2B). Overall, it seems that there is a loss of glycolysis and metabolism but an elevation of immune processes in SS tears samples. Additionally, we identified multiple proteases (Cathepsin B, MMP8, MMP9, Leukotriene A-4 hydrolase, and Prostasin) and protease inhibitors (Cystatin-C and -D) that were elevated SS tears compared to healthy controls (Table 2).

**TABLE 2:** Proteases and protease inhibitors significantly elevated in Sjogren’s Syndrome (SS) tear wash over healthy controls.

Uniprot ID	Gene name	Protein name	Fold difference SS/healthy
**Elevated in Sjogren’s Syndrome**			
P14780	*MMP9*	Matrix metalloproteinase-9	88.4
P28325	*CST5*	Cystatin-D	12.5
P22894	*MMP8*	Neutrophil collagenase	9.4
P01034	*CST3*	Cystatin-C	8.5
P07858	*CTSB*	Cathepsin B	6.2
P09960	*LTA4H*	Leukotriene A-4 hydrolase	5.3
Q16651	*PRSS8*	Prostasin	5.1
**Elevated in healthy**			
P07384	*CAPN1*	Calpain-1	−21.8
Q96KP4	*CNDP2*	Cytosolic non-specific dipeptidase	−17.7
P01042	*KNG1*	Kininogen-1	−12.4
P08697	*SERPINF2*	Alpha-2-antiplasmin	−11.9
Q99497	*PARK7*	DJ-1/Parkinson disease protein 7	−11.6
P60900	*PSMA6*	Proteasome subunit alpha type-6	−10.6
P25788	*PSMA3*	Proteasome subunit alpha type-3	−10.6
P13798	*APEH*	Acylamino-acid-releasing enzyme	−9.3
P25787	*PSMA2*	Proteasome subunit alpha type-2	−9.0
P28838	*LAP3*	Cytosol aminopeptidase	−8.5
P30086	*PEBP1*	Phosphatidylethanolamine-binding protein	−7.6
Q5JNW7	*PSMB8*	Proteasome subunit beta type-8	−6.1
P30740	*SERPINB1*	Leukocyte elastase inhibitor	−5.6

### Decrease of PRG4 in Tear Wash

**FIGURE 3:**
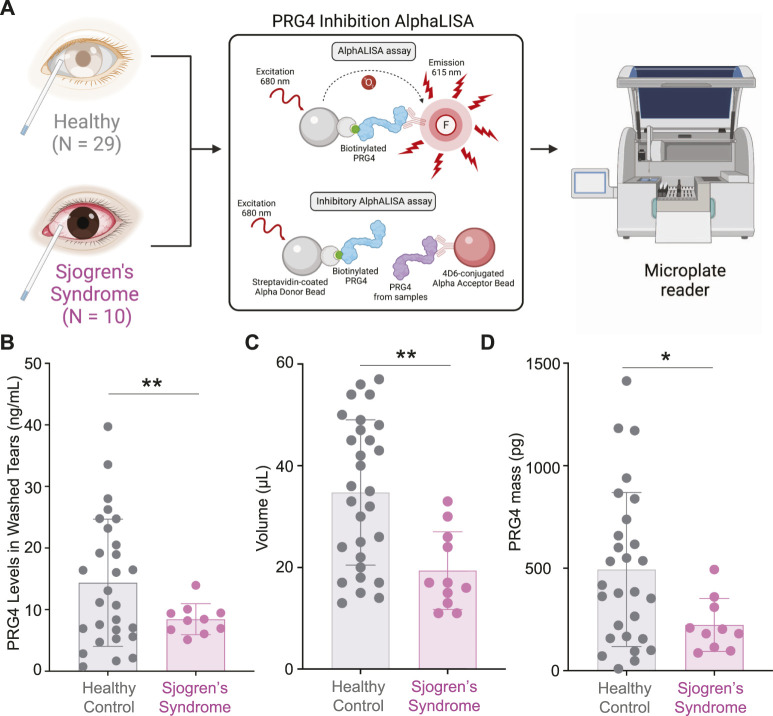
Measurements of PRG4 in Tear Washes. **(A)** AlphALISA workflow using a designed PRG4 antibody 4D6-based assay. **(B)** PRG4 levels, **(C)** volume and **(D)** PRG4 mass were measured. Data are represented as bar graphs with ±standard deviation (SD) and analyzed by a Welch’s two-tailed paired *t* test: *p > 0.05, **p > 0.01.

As we demonstrated previously, increased proteolytic activity of cathepsin S in SS tears resulted in decreased lubrication (Regmi et al., 2017) (Menon et al., 2021). Therefore, we wanted to test if the levels of PRG4, also known as lubricin, are changed between SS and healthy control tear washes. Using an AlphaLISA (Figure 3A), we measured the concentration of PRG4 in tear wash of 39 patients (healthy, N = 29 and SS, N = 10) and we found that it was significantly decreased in SS patients compared to healthy controls (Figure 3B, **p < 0.01). The collected volume of tear washes was also decreased in SS tears (Figure 3C, **p < 0.01). Therefore, the total PRG4 mass was also found to be significantly decreased in SS tears compared to healthy controls (Figure 3D, *p < 0.05). In summary, we identified a significant decrease in PRG4 levels in SS tear washes.

### Quantitative Proteomics of Sjogren’s Syndrome Saliva

**FIGURE 4:**
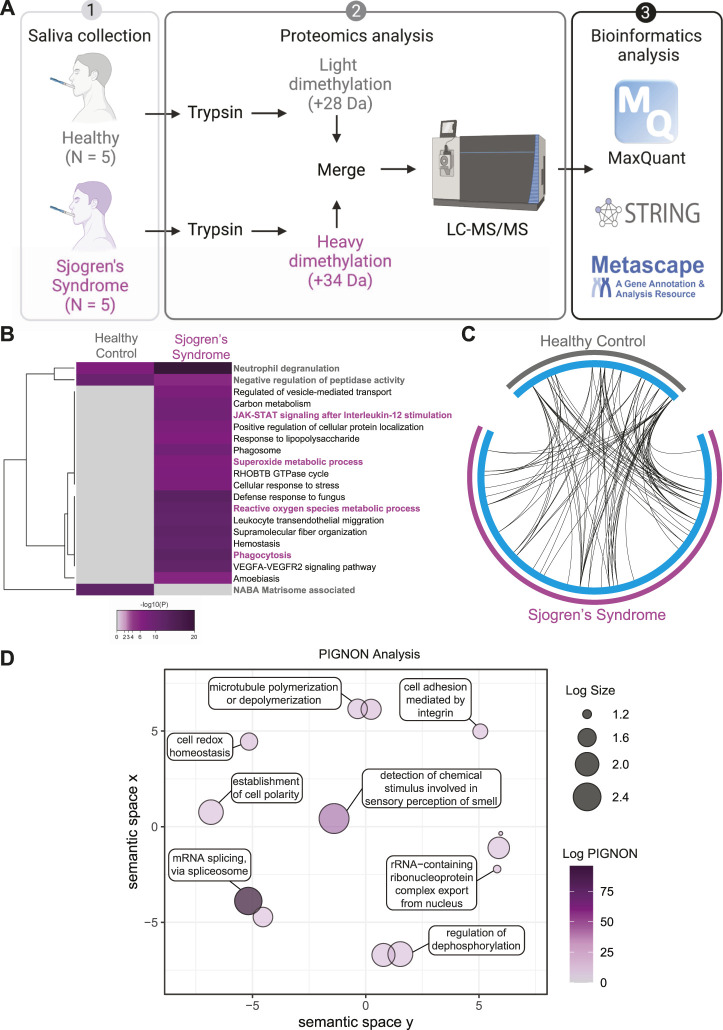
Quantitative Proteomics of Sjogren’s Syndrome Saliva. **(A)** Proteomics workflow and analysis (N = 5, Sjogren’s syndrome (SS) and N = 5 healthy controls). STRINGdb and Metascape software were used to identify pathway enrichment and protein-protein interactions. A list of all detected proteins is provided in Supplementary Table S2. **(B)** Metascape analysis of different pathways between SS and healthy control saliva samples. Magenta marked are upregulated in SS and grey marked are upregulated pathways in healthy controls. Accumulative hypergeometric *p*-values and enrichment factors were calculated and used for filtering. Remaining significant terms were then hierarchically clustered into a tree based on Kappa-statistical similarities among their genes memberships. Then, 0.3 kappa score was applied as the threshold to cast the tree into term clusters. **(C)** Circos plot shows how significant proteins from the input lists overlap and were generated with Metascape. On the outside, each arc represents the identity of each gene list (Sjogren’s syndrome in magenta and Healthy control in grey). On the inside, each cyan arc represents a gene list, where each gene has a spot on the arc. Black lines link the different genes where they fall into the same ontology term. **(D)** Visualization of PIGNON results using Revigo. The x- and *y*-axis have no intrinsic meaning. In the plot, semantically similar GO terms remain close together. LogSize equals to the Log_10_(Number or annotations for GO Term ID in *Homo sapiens* in the EBI GOA database). Log PIGNON equals to the -Log_10_(FDR-adjusted *p* value as generated by PIGNON). The PIGNON analysis was performed using the normalized protein ratios as calculated by Maxquant. PIGNON was set to use the “physical link” network (v11) from the STRING database due to the small size of our dataset. The results included mapped pathways that were trimmed according to a false discovery rate (FDR) of 1%. The lists of enriched gene ontology (GO) terms, along with the *p*-values, were submitted to Revigo to summarize the biological functions and provide visualization of enriched terms.

SS patients suffer from dry mouth (Brito-Zerón et al., 2016); therefore, we wanted to assess the global proteome changes in SS saliva compared to healthy controls (Figure 4A). Saliva lysates of 5 healthy controls and 5 SS patients were digested with trypsin and isotopically labeled with light formaldehyde (+28 Da) and heavy formaldehyde (+34 Da; Figure 4A), respectively. Data were analyzed using MaxQuant (Cox and Mann, 2008) (Cox et al., 2011) at a 1% false discovery rate (FDR), and data integration for pathway enrichment was performed with STRING-db (Szklarczyk et al., 2019), PIGNON (Nadeau et al., 2021), and Metascape (Zhou et al., 2019), which are based on reactome, GO terms (biological process), and a combination of multiple databases, respectively. For the interpretation, we describe changes in abundance of proteins as log2 fold change (SS saliva over healthy controls), which means log2 values > 0 represent proteins that were upregulated in SS tears, <0 represent downregulation. In the proteomics analysis (Supplementary Figure S3 and Supplementary Table S2), we identified 104 unique proteins that were upregulated in SS saliva and 42 unique downregulated proteins. By using Metascape, we identified several enriched pathways between SS and healthy control saliva (Figure 4B, Supplementary Figure S4A,B). Some enriched pathways in SS saliva include JAK-STAT signaling after interleukin-12 stimulation (CTSG, ITGB2, ITGAM, LCP1, TALDO1), superoxide metabolic process (CBR1, MPO, ARG1, RYR2, ITGAM) and phagocytosis (CTSG, ELANE, ITGAM, ITGB2, PRG4, PRTN3, MPO, PARK7) (Figure 4B, Supplementary Table S2). Conversely, some enriched pathways in the healthy control saliva include neutrophil degranulation (CD14, MMP8, S100A11, SLPI), negative regulation of peptidase activity (ECM1, CST1, CST4, CST5, SLPI, SERPINB3, TIMP1) and NABA matrisome associated (FLG, FLG2, MMP8, MUC5B, MUC7, RPTN, TIMP1) (Figure 4B, Supplementary Table S2). Although we identified key differences in pathway enrichment and GO terms, there was some overlap between the SS and healthy control saliva (Figure 4C).

**FIGURE 5:**
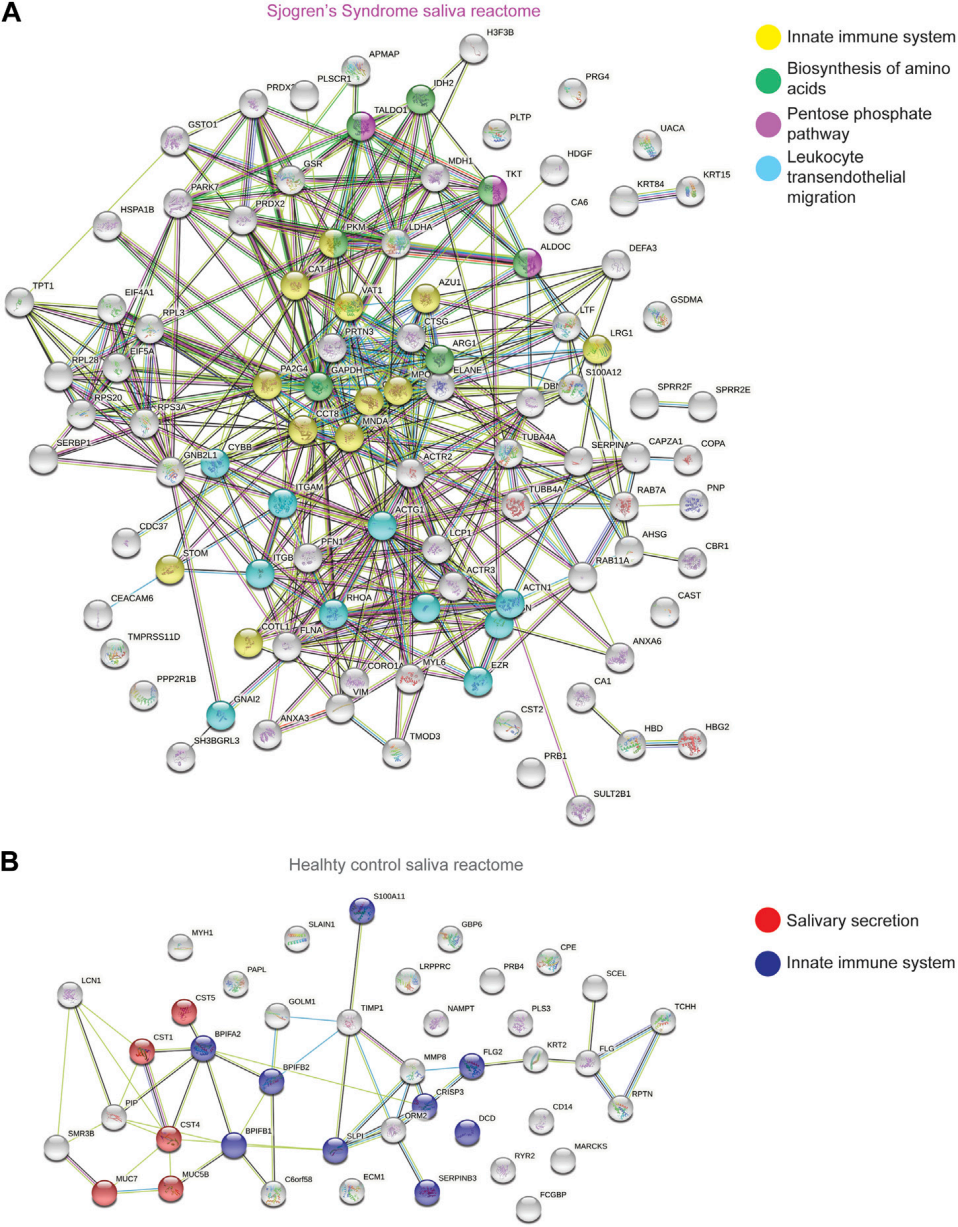
STRING Analysis of Quantitative Proteomics Data of Human Sjogren’s Syndrome Saliva Samples. Analysis of protein−protein interaction network by STRING v11. Elevated proteins from **(A)** SS or **(B)** healthy control saliva samples identified from the quantitative proteomics analysis were mapped by searching the STRING v11 software with a confidence level of 1% false discovery rate. Colored lines between the proteins indicate different types of interaction evidence: known interactions (teal), experimentally determined (pink), predicted interactions gene neighborhood (green), gene fusions (red), gene co-occurrence (blue), text-mining (yellow), coexpression (black), protein homology (purple).

To better understand and visualize our data, we used two additional bioinformatics tools, PIGNON (Nadeau et al., 2021) and STRING-db (Szklarczyk et al., 2019), to add a more comprehensive characterization of our tear datasets. Using PIGNON, we identified mRNA splicing (*via* spliceosome), cell adhesion mediated by integrin and detection of chemical organization stimulus involved in sensory perception of smell (Figure 4D, Supplementary Figure S4C). Using STRING-db v11 to analyze the reactome pathways, we observed that the majority of the upregulated proteins in SS saliva were innate immune system as yellow nodes (AZU1, LRG1, MPO, STOM, VAT1), biosynthesis of amino acids as green nodes (ALDOC, ARG1, IDH2, PKM, TKT), pentose phosphate pathway as purple nodes (ALDOC, TALDO1, TKT) and leukocyte transendothelial migration as cyan nodes (ACTN1, ITGAM, ITGB2, RHOA) reactome pathways (Figure 5A). In healthy control saliva, enriched reactome pathways included proteins clustered with salivary secretion as red nodes (CST1, CST4, CST5, MUC5B, MUC7) and innate immune system as blue nodes (BPIFA2, BPIFB1, BPIFB2, DCD, S100A11, SERPINB3, SLPI) (Figure 5B). Interestingly, using quantitative proteomics, we identified a 2.4-fold increase of PRG4 in SS saliva. Additionally, we identified multiple proteases and protease inhibitors that had various expression levels between SS and healthy control saliva (Table 3). As expected, our analysis of tears and saliva samples resulted in the identification of different proteins that are likely contributing in distinct ways to the pathogenesis of SS in a fluid/tissue-dependent manner (Supplementary Figure S5).

**TABLE 3:** Proteases and protease inhibitors significantly elevated in Sjogren’s Syndrome (SS) saliva over healthy controls.

Uniprot ID	Gene name	Protein name	Fold difference SS/healthy
**Elevated in Sjogren’s Syndrome**			
Q99497	*PARK7*	DJ-1/Parkinson disease protein 7	3.9
P08311	*CTSG*	Cathepsin G	3.8
P08246	*ELANE*	Neutrophil elastase	3.6
P02788	*LTF*	Lactransferrin	3.6
P20160	*AZU1*	Azurocidin	3.3
P09228	*CST2*	Cystatin-SA	3.1
P20810	*CAST*	Calpastatin	3.1
P24158	*PRTN3*	Proteinase 3/Myeloblastin	3.0
P01009	*SERPINA1*	Alpha-1-antitrypsin	2.9
O60235	*TMPRSS11D*	Transmembrane protease serine 11D	2.5
***Elevated in healthy***			
P16870	*CPE*	Carboxypeptidase We	-4.0
Q9HC84	*MUC5B*	Mucin-5B	−3.4
P01037	*CST1*	Cystatin-SN	−3.2
P28325	*CST5*	Cystatin-D	−2.8
P01036	*CST4*	Cystatin-S	−2.6
P22894	*MMP8*	Neutrophil collagenase	−2.5
P29508	*SERPINB3*	Serpin B3	−2.5
P03973	*SLPI*	Antileukoproteinase	−2.0

### Increase of PRG4 in Saliva and Parotid Gland

**FIGURE 6:**
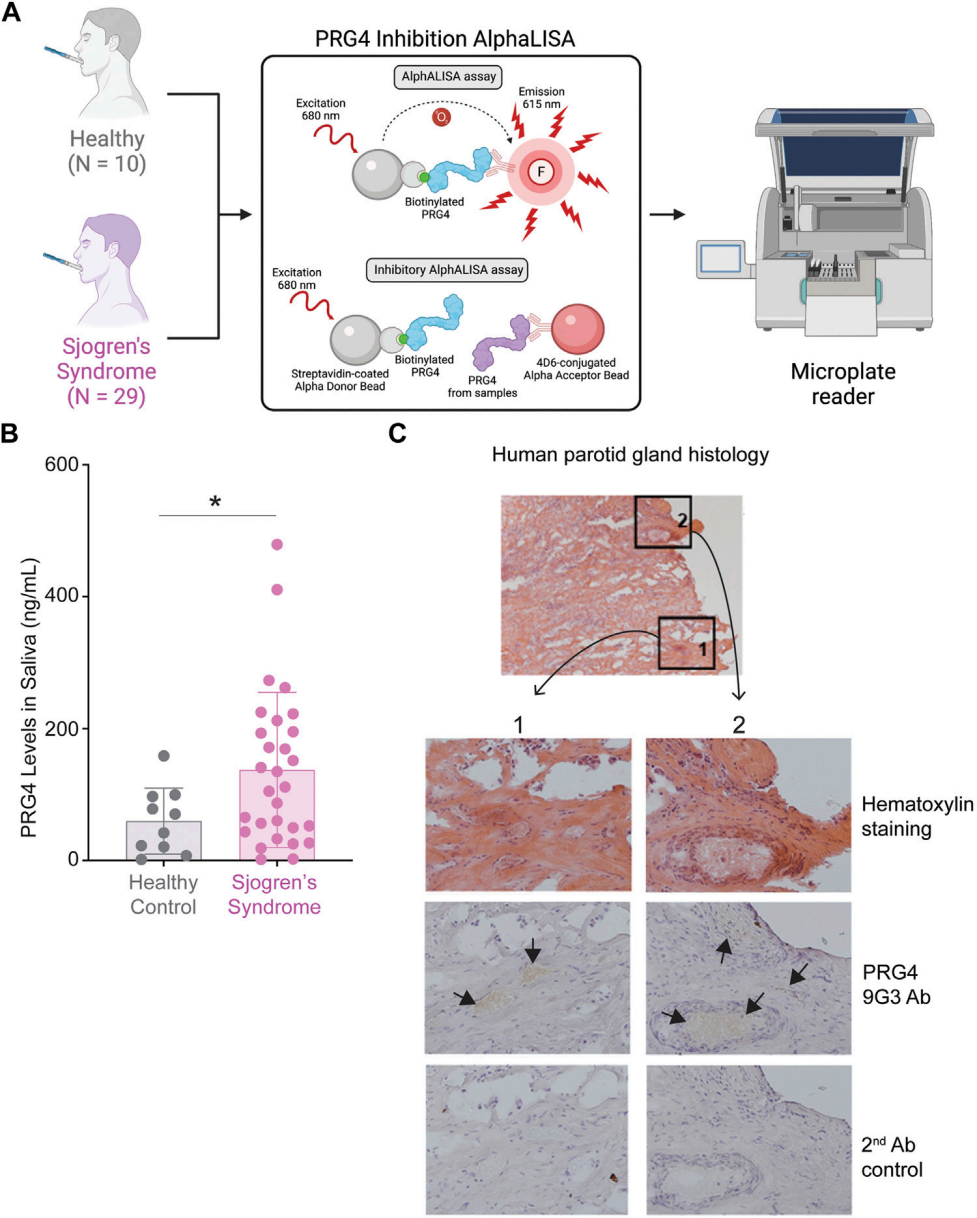
increase of PRG4 in Saliva and Parotid Gland. **(A)** AlphALISA workflow using a designed PRG4 antibody 4D6-based assay. **(B)** PRG4 levels were measured. Data are represented as bar graphs with ±standard deviation (SD) and analyzed by a Welch’s two-tailed paired *t* test: *p > 0.05, **p > 0.01. **(C)** Parotid gland histology using hematoxylin staining and a PRG4 antibody (9G3). As a control, secondary antibody control was used without the addition of a primary PRG4 antibody.

As we identified a 2.4-fold increase of PRG4 in SS saliva (Supplementary Table S2), we wanted to validate our finding using an AlphALISA (Figure 6A). We measured the concentration of PRG4 in saliva of 39 patients (healthy, N = 10 and SS, N = 29) and we found a significant difference of PRG4, corresponding to a 2.3-fold increase in SS patients compared to healthy controls (Figure 6B, *p < 0.05). Another commons symptom of SS is salivary glands swelling, as the parotid glands are commonly involved in SS (Rischmueller et al., 2016); therefore, we wanted to verify the PRG4 expression level. By analyzing five different blocks from 4 different patients, PRG4 signal was found in both the serous acinii and the striated duct (Figure 6C), indicating that PRG4 is produced and secreted by the parotid gland. This signal was specific, as shown by the lack of signal in the secondary only control (Figure 6C).

## Discussion

To improve current diagnosis and therapeutic responses in SS patients, better characterization and understanding of the disease is urgently needed. With a coherent pipeline connecting potential biomarker identification with well-established methods for validation, mass spectrometry-based approaches are an effective way to discover novel biomarkers (Rifai et al., 2006; Aebersold and Mann, 2016; Nakayasu et al., 2021). Identifying protein biomarkers in tear washes and saliva offers several advantages as it is manageable for sampling/repeated sampling in addition to being inexpensive and non-invasive (Chen et al., 2015; Nakayasu et al., 2021). Here, we performed an unbiased investigation using quantitative proteomics of SS saliva and tear washes compared to healthy controls. In tear washes, we identified significant changes in proteins expression associated with leukocyte transendothelial migration, which is an early inflammatory event occurring before the differentiation and activation of leukocytes and a subsequent downstream cascade of immune and inflammatory signaling (Muller, 2011). Changes in the transendothelial migration of leukocyte pathway is a feature of other inflammatory diseases like SLE (Marshall, 2003) or atherosclerosis (Shang et al., 2014). In the MRL/lpr autoimmune model in mouse that resembles human SLE, intercellular adhesion molecule 1 (ICAM-1) dependent exaggerated leukocyte-endothelial interactions was found to be associated with disease onset and severity (Marshall, 2003). Previous studies had characterized the transendothelial migration of leukocytes, as per the analysis of gene expression profiles of mRNA from parotid glands of SS patients (Wei et al., 2018). Using quantitative proteomics, we also identified an enrichment of proteins associated with transendothelial migration of leukocyte associated with an elevation of cathepsin B (CTSB), integrin beta 2 (ITGB2) and matrix metalloproteinase-9 (MMP9) in SS tear wash. Higher expression of MMP9 in tears aligns with previous findings which reported that an increase expression of MMP9 in tears was correlated with increased disease severity in patients with dry eye disease (Luo et al., 2004; Pinto-Fraga et al., 2018) and also in SS patients’ saliva (Garreto et al., 2021). Cathepsin S has been previously demonstrated to be elevated in SS tears (Hamm-Alvarez et al., 2014; Regmi et al., 2017; Hargreaves et al., 2019). In our study, we identified an elevation of cathepsin B in SS tears, which was previously demonstrated in the first 5 years of SS diagnosis (Sohar et al., 2005). Pharmacological inhibition of cathepsin B was shown to reverse ICAM-1 dependent leukocyte endothelial adhesion in neurovascular inflammation (Wang et al., 2019). A better characterization of the role of cathepsin B in SS could reveal a potential approach to reduce vascular inflammation in the eye. Proteins regulating leukocyte functions, several integrins, have been drug targets for autoimmune diseases such as psoriasis and multiple sclerosis; however, a better understanding of their individual functions could result in better treatment strategies and avoid side effects associated with integrin inhibitions such as progressive multifocal leukoenphalopathy (Mitroulis et al., 2015) (Chopra et al., 2021). We also identified an elevation of the protein-lipid complex remodeling pathway associated with apolipoprotein E (APOE) in SS tears. APOE was associated with preventing disease progression in the experimental autoimmune encephalomyelitis (EAE) mouse model by regulating Th1 and Th17 responses (Wei et al., 2013). Interestingly, in SS, APOE polymorphisms were significantly associated with the early onset of disease (Pertovaara, 2004). Additionally, although the patients we evaluated here did not have cancer, SS patients are more susceptible to cancer development (Brito-Zerón et al., 2016; Igoe et al., 2020); yet, the specific reason is not known but sustained and increased inflammation observed in SS patients could be associated with increased cancer risk. The expression of cystatin S (CTS3) was reported to be significantly elevated in saliva from SS patients (van der Reijden et al., 1996) and here we found a potential association of cystatin S with collagen catabolic process, neutrophils and immune system enrichment pathways. MMP8 levels were elevated in SS tear wash and were previously found to be associated with ocular inflammation, as its expression in tears was significantly higher in patients with ocular rosacea compared to healthy controls (Määttä et al., 2006).

Downregulation of glycolysis in SS tear wash compared to healthy controls indicated a dysregulation of metabolism in SS. We identified other dysregulated pathways associated with metabolism between healthy and SS saliva; specifically, the biosynthesis of amino acids and pentose phosphate pathways were upregulated in SS saliva compared to healthy controls. In inflammation, pentose phosphate pathway was demonstrated to act as a key gatekeeper by supplying ribose-5-phophate to increase cell proliferation and NADPH for antioxidative defense (Perl, 2017). Metabolic profiling of saliva demonstrated differential expression of metabolites in SS compared to healthy controls (Mikkonen, 2012). Analysis of blood serum revealed that SS patients tend to have a higher prevalence of metabolic disorders, such as dyslipidemia, diabetes mellitus and hyperuricemia, in comparison with an age and sex-matched control group (Ramos-Casals et al., 2007). Therapeutic approaches targeting metabolic pathways, such as the mTOR pathway, have been suggested as a way to treat SLE (Perl, 2017). However, no unbiased metabolomics profiling has yet been performed on SS tear or saliva samples and relatively little is known about what metabolites are implicated in SS pathogenesis.

*In vivo* pre-clinical animal studies and *ex vivo* studies on human tissues have demonstrated that altered expression and function of PRG4 (i.e. diminished lubrication and increased inflammation) has been associated with joint damage and pain in osteoarthritis (OA) and RA (Ludwig et al., 2012; Iqbal et al., 2016; Das et al., 2019). We previously demonstrated that *ex vivo* proteolytic processing of PRG4 in tears reduced boundary lubricating ability compared to unprocessed controls (Regmi et al., 2017) and that proinflammatory cytokines altered the expression of PRG4 by corneal epithelium cells *in vitro* (Menon et al., 2021)*.* Here, we show, for the first time, that PRG4 has different levels in SS tear wash and saliva compared to healthy controls. In SS tear washes, we found reduced concentration, tear volume, and total mass of PRG4 compared to healthy controls. This could be due to increased degradation from CTSS (Regmi et al., 2017) or CTSB (Elsaid et al., 2007) which was upregulated in our quantitative proteomics data. Although a previous study on PRG4 levels in the SS tears had found no significant changes between the SS and healthy controls (Schmidt et al., 2018), the AlphaLISA used in that study was based on an anti-PRG4 mAb 9G3-coated Protein G bead, which had previously been shown to be effective in measuring PRG4 levels *ex vivo* in pericardial and synovial fluid (Park et al., 2018). However, the protein-G beads were later found to interact with immunoglobulin G (IgG) molecules present in tears and as IgGs have been shown to be elevated in SS, it made the interpretation difficult (Domingo et al., 1998). Thus, the 9G3-based AlphaLISA can still be an effective tool for measuring biological samples where the concentration of IgG is low; however, an optimized experimental setup was required to measure PRG4 in tears and saliva. Thus, our newly designed 4D6-based assay did not react with sample IgGs, making our PRG4 measurements accurate. One limitation of our study was that our proteomics analysis was performed on neat tears while our PRG4 analysis was performed on tear washes. The samples analyzed by quantitative proteomics (Figure 1A) were previously analyzed using the 9G3-based AlphaLISA (Schmidt et al., 2018) but there was not enough sample volume to re-analyze them using the newly developed 4D6-based AlphaLISA. Since SS patients typically produce a significantly lower volume of tear fluid than healthy controls, analyzing tear washes allows for a greater variety of testing methods while still preserving the qualities of the original tear fluid. Future work could explore if the trends found in tear washes also apply to basal tears. The presence of PRG4 staining in both the serous acini, where proteins are typically secreted, and the striated duct, which directly leads to the interlobular ducts where saliva is secreted, indicates that PRG4 is produced and secreted into the oral cavity. Interestingly, a 2.3- and 2.4-fold increase of PRG4 in SS saliva concentration was found, as measured by AlphALISA and quantitative proteomics, respectively. However, we identified a decrease of PRG4 in tear samples. A possible explanation could be that the body responds to normalize salivary homeostasis. Another explanation could be that endogenous PRG4 is more concentrated in a lower volume since less saliva is produced by SS patients. Future studies could examine how PRG4 production and secretion in the salivary gland is influenced by SS.

Our metascape analysis identified that the JAK-STAT signaling after interleukin-12 stimulation was enriched in SS saliva compared to healthy controls. In a cell culture system using the human salivary gland (HSG) cell line, treatment with JAK inhibitors (AG490 and ruxolitinib) resulted in suppression of the innate epigenetic reprogramming observed when these cells were treated with interferon-α (IFNα), IFNγ and H_2_O_2_ to re-create a cellular model of proinflammation (Charras et al., 2020). In preclinical models of RA and EAE, JAK-STAT inhibition was demonstrated to be effective by suppressing proinflammatory cytokines and chemokines (Stump et al., 2011) (Liu et al., 2014). We detected an enrichment in pathways associated with polymorphonuclear leukocytes as indicated by myeloperoxidase (MPO) in SS tear and saliva. While the potential role of oxidative stress in ocular surface inflammation and dry eye disease has been investigated (Dogru et al., 2018), our study suggests an upregulation of superoxide metabolic processes in the saliva of patients with SS. Aberrant phagocytosis in the peripheral blood of patients with primary SS has also been reported (Fragoulis et al., 2015). Our analyses have also identified an upregulation of phagocytosis in SS saliva compared to healthy controls.

There are a few limitations of our current study. Collecting enough proteins from tears and saliva to run quantitative proteomics can be a challenge. Therefore, additional samples would need to be run in the future to assess additional factors such as sex differences (as SS is more prevalent in women than men), age and drugs that the patients are taking and see if they impact certain significant proteins that we have detected in addition to proteases, protease inhibitors and PRG4. Our current proteomic analysis has revealed enrichment of several newly associated but also previously reported proteases and protease inhibitors in SS. The activity of cysteine proteases (cathepsins) and serine proteases (neutrophil elastase, prostasin, proteinase-3/myeloblastin) could be further investigated using activity-based probes in SS (Edgington-Mitchell et al., 2017; Anderson et al., 2019, 2020; Mainoli et al., 2020; Mountford et al., 2020; Tu et al., 2021). The upregulation of cathepsin G in the saliva of patients with SS is likely associated with an increase in neutrophils and corresponds to an elevation in synovial fluid of RA patients (Gao, 2018). Calpain-1 was upregulated in healthy control tears and was previously found to play an important role in cerebellar plasticity and eye-blink conditioning, as evidenced by impairment in both of these measures in *Capn1* ^*−/−*^ mice (Heysieattalab et al., 2020). Interestingly, upregulation of several cysteine protease inhibitors in the saliva of healthy controls included cystatin-D, cystatin-S and cystatin-SN, indicating a possible regulation and inhibition of cathepsin activity (Balbín et al., 1994). Further studies to characterize these proteases enriched in SS tears and saliva using N-terminomics could identify their potential substrates, which could help find new drug targets in SS (Dufour, 2015; Mainoli et al., 2020; Longxiang Wang, Kimberly Main, Henry; Wang, Olivier Julien, 2021). Our data present new proteases, metabolic and cellular signaling pathways that are elevated in SS and could lead to unbiased investigations of new regulators of SS pathogenesis.

## Data Availability

The original contributions presented in the study are publicly available. This data can be found here *via* the PRIDE database and are freely available: PXD028922.
