# Supplementary material for: Proteomics Analysis of Tears and Saliva From Sjogren’s Syndrome Patients
Source: Front Pharmacol. 2021 Dec 7;12:787193. doi: 10.3389/fphar.2021.787193 (PMC8689002; doi:10.3389/fphar.2021.787193)
Supplement: Supplementary file 1 [file DataSheet2.PDF]

Supplementary Figure 1

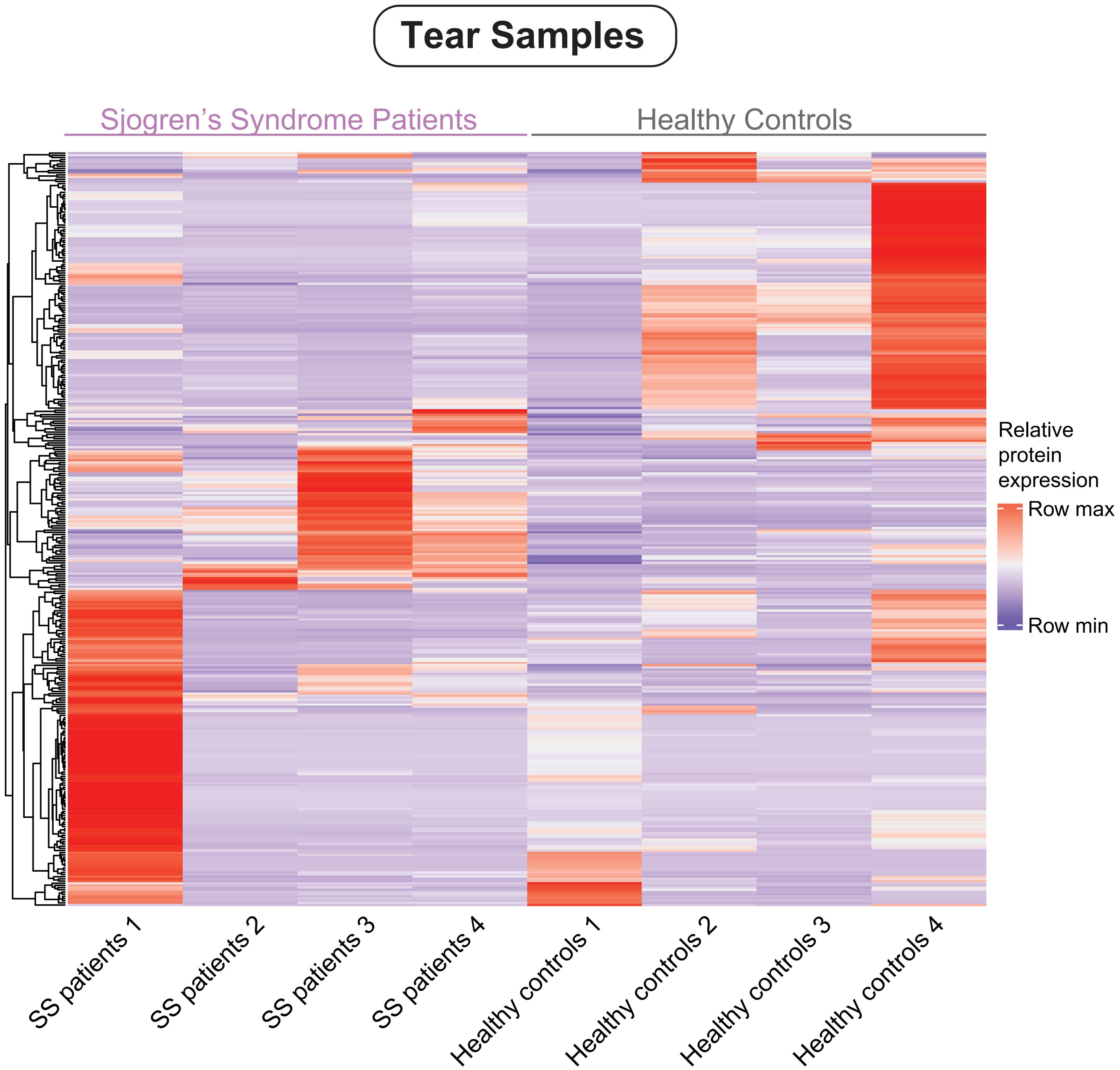

Supplementary Figure 1: Heatmap of the tear proteomic analysis. Intensity values were scaled and centered for each protein. The protein quantifications are shown for 4 pooled patients' samples.
