# Supplementary material for: Proteomics Analysis of Tears and Saliva From Sjogren’s Syndrome Patients
Source: Front Pharmacol. 2021 Dec 7;12:787193. doi: 10.3389/fphar.2021.787193 (PMC8689002; doi:10.3389/fphar.2021.787193)
Supplement: Supplementary file 3 [file DataSheet6.PDF]

Supplementary Figure 5

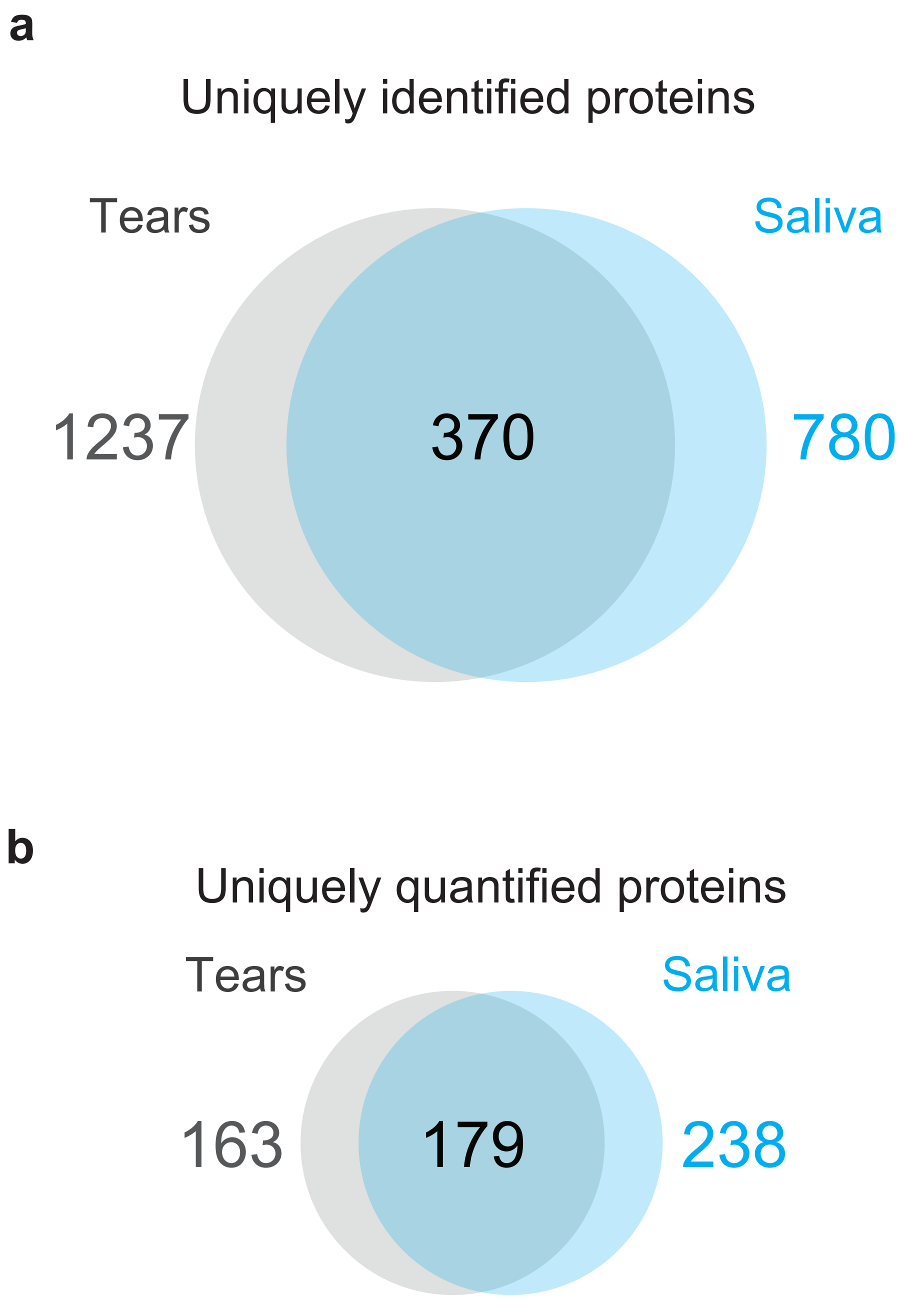

Supplementary Figure 5: Venn diagrams of **a)** uniquely identified proteins and **b)** uniquely quantified proteins between tears and saliva proteomics analyses.
