# Supplementary material for: Proteomics Analysis of Tears and Saliva From Sjogren’s Syndrome Patients
Source: Front Pharmacol. 2021 Dec 7;12:787193. doi: 10.3389/fphar.2021.787193 (PMC8689002; doi:10.3389/fphar.2021.787193)
Supplement: Supplementary file 4 [file DataSheet7.docx]

**Supplementary information**: Individual patient’s information.

**Tears:** Individual patient’s information (sex and age).

|  | **Sex** | **Age** |
| --- | --- | --- |
| **Healthy** | Female | 20 |
|  | Female | 21 |
|  | Female | 22 |
|  | Female | 23 |
|  | Female | 23 |
|  | Female | 23 |
|  | Female | 24 |
|  | Female | 24 |
|  | Female | 27 |
|  | Female | 29 |
|  | Female | 52 |
|  | Female | 56 |
|  | Female | 59 |
|  | Male | 24 |
|  | Male | 28 |
|  | Male | 31 |
|  | Male | 31 |
|  | Male | 31 |
|  | Male | 36 |
|  | Male | 39 |
| **Sjogren’s Syndrome**  **(SS)** | Female | 26 |
|  | Female | 29 |
|  | Female | 40 |
|  | Female | 46 |
|  | Female | 51 |
|  | Female | 53 |
|  | Female | 57 |
|  | Female | 57 |
|  | Female | 59 |
|  | Female | 62 |
|  | Female | 64 |
|  | Female | 67 |
|  | Female | 69 |
|  | Female | 72 |
|  | Female | 74 |
|  | Female | 75 |
|  | Female | 75 |
|  | Female | 77 |
|  | Female | 77 |
|  | Female | 78 |
|  | Male | 31 |
|  | Male | 81 |
|  | | |
| Healthy avg | 13 females, 7 males | 31.2 ± 11.4 years old |
| SS avg | 15 females, 2 males | 60.0 ± 16.5 years old |

**Tear Washes:** Individual patient’s information (sex and age).

|  | **Sex** | **Age** |
| --- | --- | --- |
| **Healthy** | Female | 18 |
|  | Female | 21 |
|  | Female | 21 |
|  | Female | 21 |
|  | Female | 22 |
|  | Female | 23 |
|  | Female | 23 |
|  | Female | 27 |
|  | Female | 27 |
|  | Female | 41 |
|  | Female | 42 |
|  | Female | 42 |
|  | Female | 43 |
|  | Female | 43 |
|  | Female | 48 |
|  | Female | 66 |
|  | Male | 19 |
|  | Male | 19 |
|  | Male | 19 |
|  | Male | 19 |
|  | Male | 23 |
|  | Male | 30 |
|  | Male | 37 |
|  | Male | 42 |
|  | Male | 44 |
|  | Male | 46 |
|  | Male | 56 |
|  | Male | 64 |
| **Sjogren’s Syndrome**  **(SS)** | Female | 36 |
|  | Female | 46 |
|  | Female | 50 |
|  | Female | 51 |
|  | Female | 51 |
|  | Female | 59 |
|  | Female | 59 |
|  | Female | 61 |
|  | Female | 64 |
|  | Female | 64 |
|  | Female | 65 |
|  | Female | 77 |
|  | Female | 80 |
|  | Male | 70 |
|  | | |
| Healthy avg | 17 females, 12 males | 34.1 ± 14.2 years old |
| SS avg | 13 females, 1 male | 59.5 ± 12.0 years old |

**Saliva:** Individual patient’s information (sex and age).

|  | **Sex** | **Age** |
| --- | --- | --- |
| **Healthy** | Female | 36 |
|  | Female | 37 |
|  | Female | 55 |
|  | Female | 63 |
|  | Female | 63 |
|  | Male | 22 |
|  | Male | 31 |
|  | Male | 47 |
|  | Male | 55 |
|  | Male | 59 |
| **Sjogren’s Syndrome**  **(SS)** | Female | 22 |
|  | Female | 31 |
|  | Female | 31 |
|  | Female | 31 |
|  | Female | 32 |
|  | Female | 32 |
|  | Female | 35 |
|  | Female | 36 |
|  | Female | 36 |
|  | Female | 38 |
|  | Female | 39 |
|  | Female | 42 |
|  | Female | 43 |
|  | Female | 44 |
|  | Female | 47 |
|  | Female | 49 |
|  | Female | 50 |
|  | Female | 54 |
|  | Female | 54 |
|  | Female | 58 |
|  | Female | 74 |
|  | Female | 87 |
|  | Male | 31 |
|  | Male | 34 |
|  | Male | 45 |
|  | Male | 45 |
|  | Male | 46 |
|  | Male | 59 |
|  | Male | 63 |
|  | Male | 68 |
|  | | |
| Healthy avg | 5 females, 5 males | 46.8 ± 14.5 years old |
| SS avg | 13 females, 1 male | 45.2 ± 14.6 years old |
