# Supplementary material for: Proteomics Analysis of Tears and Saliva From Sjogren’s Syndrome Patients
Source: Front Pharmacol. 2021 Dec 7;12:787193. doi: 10.3389/fphar.2021.787193 (PMC8689002; doi:10.3389/fphar.2021.787193)
Supplement: Supplementary file 6 [file Image4.pdf]

Supplementary Figure 4

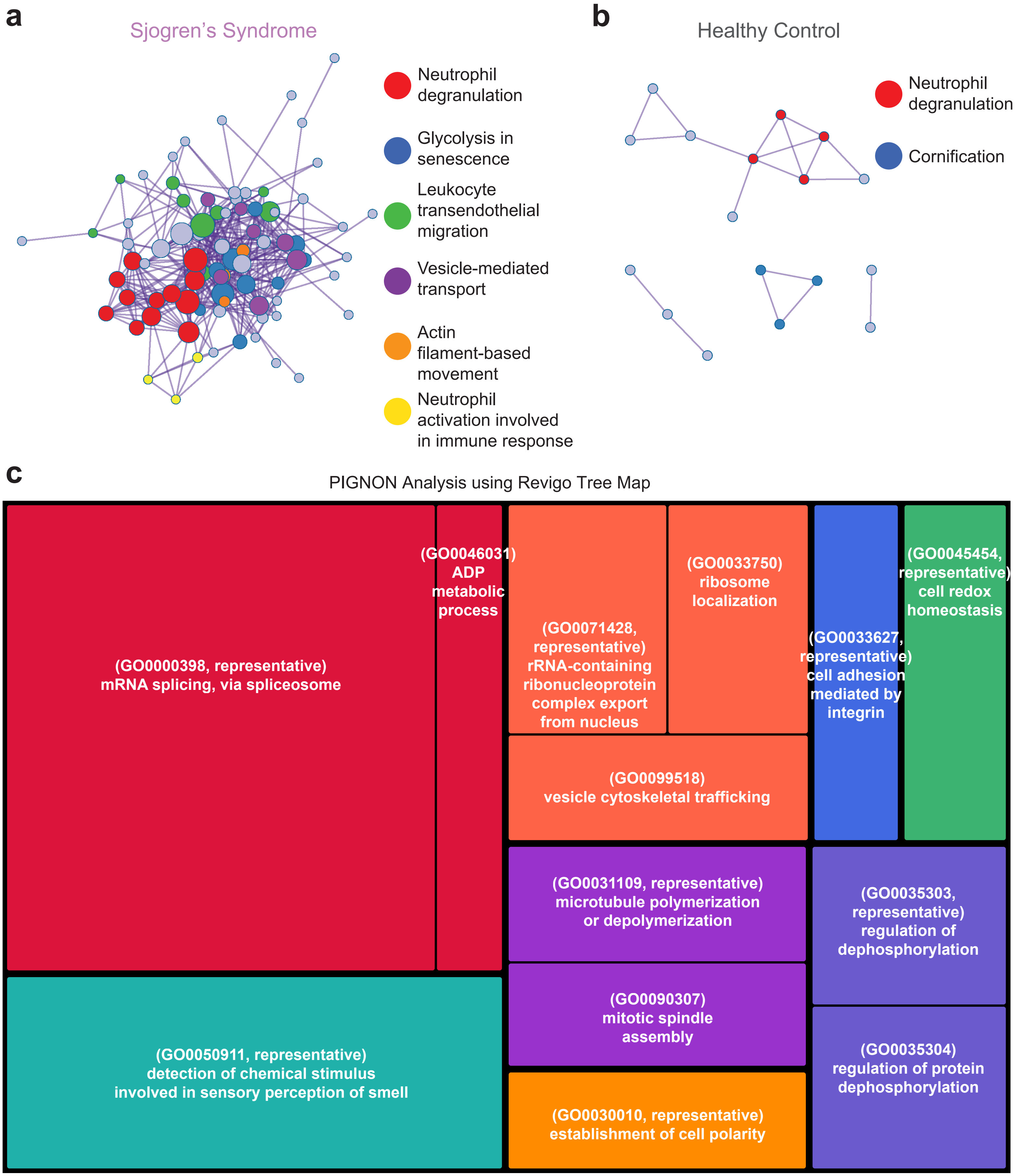

Supplementary Figure 4: Metascape analysis of **a)** Sjogren's syndrome and **b)** Healthy Control saliva. All protein-protein interactions (PPI) among each input gene list were extracted from PPI data source and formed a PPI network. Gene Ontology (GO) enrichment analysis was applied to the network to assign biological "meanings". **c)** GO terms identigied using PIGNON analysis and drawn using Revigo.
